# A Case of Lemierre Syndrome Secondary to Otitis Media and Mastoiditis

**DOI:** 10.1155/2014/208960

**Published:** 2014-11-06

**Authors:** Aynur Turan, Harun Cam, Yeliz Dadali, Serdar Korkmaz, Ali Özdek, Baki Hekimoğlu

**Affiliations:** ^1^Department of Radiology, Diskapi Yildirim Beyazit Training and Research Hospital, Etlik, 06010 Ankara, Turkey; ^2^Department of Radiology, Faculty of Medicine, Ahi Evran University, Kirşehir, Turkey; ^3^Department of Otolaryngology, Diskapi Yildirim Beyazit Training and Research Hospital, Ankara, Turkey

## Abstract

Lemierre's syndrome is a rare clinical condition that generally develops secondary to oropharyngeal infection caused by *Fusobacterium necrophorum*, which is an anaerobic bacteria. A 62-year-old patient with diabetes mellitus presented with internal jugular vein and sigmoid sinus-transverse sinus thrombophlebitis, accompanying otitis media and mastoiditis that developed after an upper airway infection. Interestingly, there were air bubbles in both the internal jugular vein and transverse sinus. Vancomycin and meropenem were started and a right radical mastoidectomy was performed. The patient's clinical picture completely resolved in 14 days. High mortality and morbidity may be prevented with a prompt diagnosis of Lemierre's syndrome.

## 1. Introduction

In 90% of cases, the cause of Lemierre's syndrome (LS) is the* Fusobacterium necrophorum*, which is an anaerobic, gram negative bacteria. On the other hand, anaerobic* Streptococci* and other gram negative anaerobic bacteria are responsible for the remaining 10% of cases [1]. The disease has been described in detail by Lemierre in 1936 [2]. Mortality rates were as high as 90% in its first described periods and decreased to 4–18% with the widespread use of antibiotics [2, 3]. The current study aimed to draw attention to this disease described as the “forgotten disease” in the literature [4] and present the radiological findings of a case of internal jugular vein (IJV) thrombophlebitis and accompanying sigmoid-transverse sinus thrombosis that was believed to develop secondary to otitis media (OM) and mastoiditis.

## 2. Case

**Figure 1:**
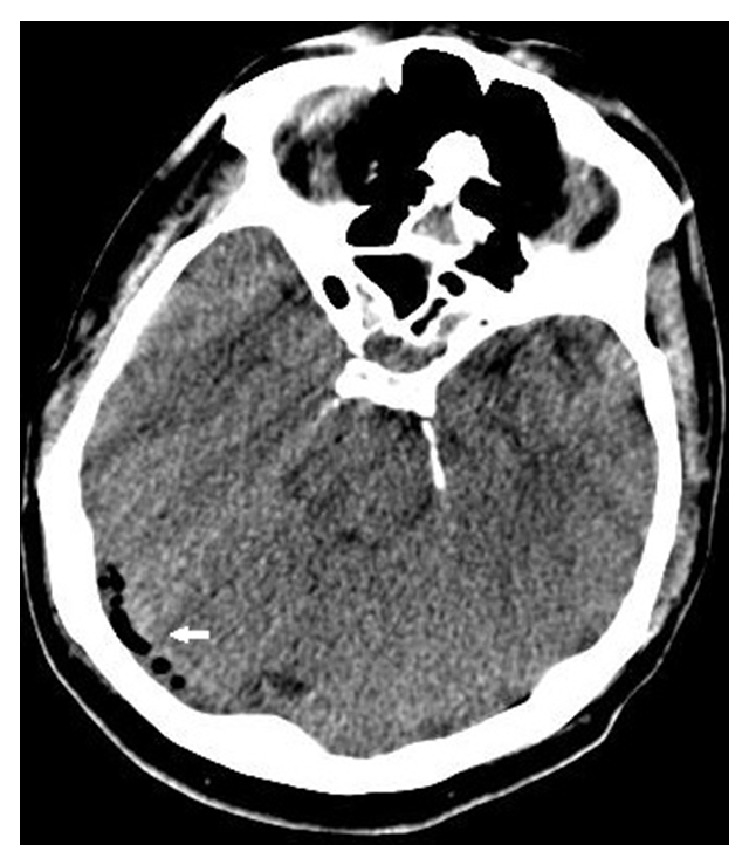
Air bubbles as seen in the brain CT of the right sigmoid sinus.

**Figure 2:**
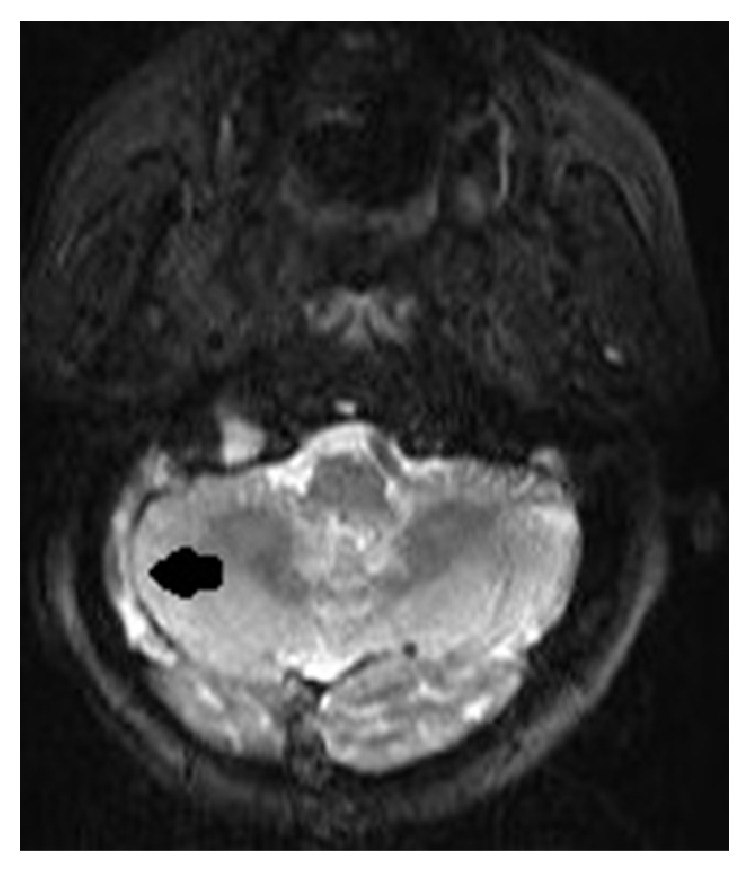
Axial T2-weighted MRI revealed signal void loss on the right sigmoid and transverse sinuses.

**Figure 3:**
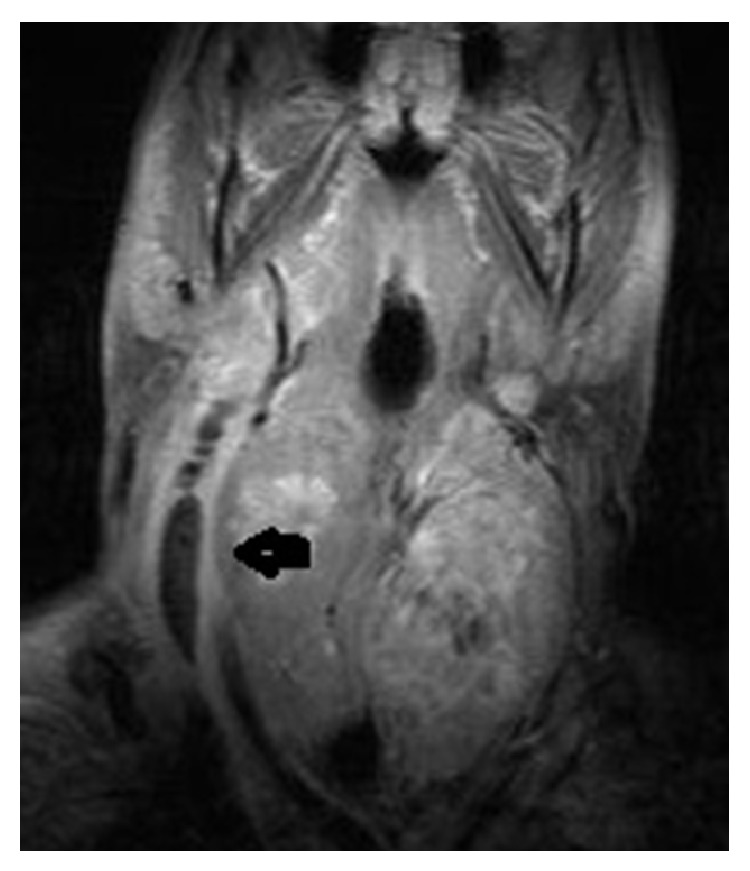
Neck MRI with contrast showed internal jugular venous distention with a thickened enhancing wall and filling defects.

**Figure 4:**
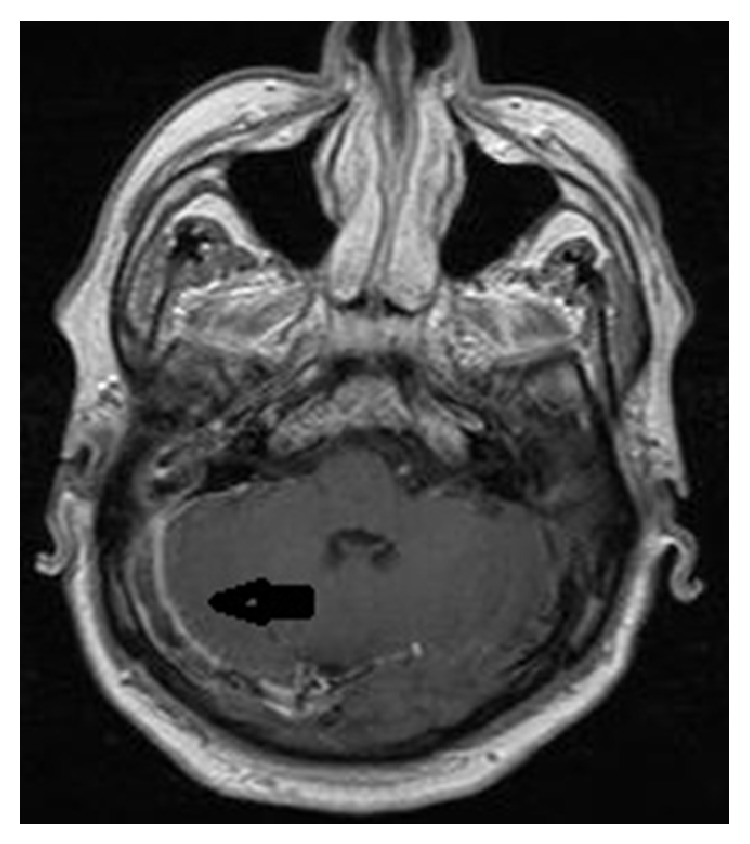
Axial brain MRI with contrast showed filling defects in right sigmoid and transverse sinuses.

A 62-year-old male patient was on cefuroxime axetil treatment for an upper airway infection that persisted for 15 days. However, he was admitted to the emergency department with complaints of redness, swelling, and pain increasing with motion in the neck region, leakage from the right ear, headache, shivering, and fever. His body temperature was 39°C upon physical examination. There was grade I tonsillar hypertrophy in the oropharyngeal examination. The right external ear way was edematous and the tympanic membrane was macerated. There was also tenderness in the neck region. Any abnormalities related to the lungs were not present in the physical examination and the neurological examination was normal. No other additional findings were detected in the physical examination. The patient had a history of diabetes mellitus type 2 (T2DM) for 15 years. His blood glucose was 317 mg/dL and hemoglobin A1c was 10.2%, indicating poorly controlled T2DM. His white cell count was 18,000/mm^3^ and other pertinent laboratory results were unremarkable. With the clinical suspicion of deep neck infection, brain and neck computed tomography (CT) was performed. The neck CT revealed internal jugular venous distention with a thickened enhancing wall, filling defects in the lumen, and air bubbles. In the brain CT, there were aeration defects and effusion on mastoid cellules and the middle ear and filling defects and air bubbles in the right sigmoid and transvers sinuses (Figure 1). In order to determine the extent and other accompanying complications, contrasted brain and neck MRI were performed, which revealed that there was signal void loss in T2WI on the right IJV (Figure 2), right sigmoid, and transverse sinus; internal jugular venous distention with a thickened enhancing wall and filling defects in the IJV and sigmoid-transverse sinus lumens (Figures 3 and 4). Thrombosis of other intracerebral veins was not observed. Moreover, there were inflammatory signal changes on the right mastoid cellules and the middle ear cavity. These findings were evaluated as compatible with right OM, mastoiditis, IJV, sigmoid, and transverse sinus thrombophlebitis. Air bubbles observed both in the IJV and transverse sinus alluded to the fact that the infection was caused by anaerobe bacteria. The blood culture was negative. The patient was diagnosed with Lemierre's syndrome, owing to the OM and mastoiditis and accompanying sigmoid and transverse sinus thrombosis. Vancomycin 2 gr/day IV and Meropenem 3 × 2 gr/day IV treatments were administered for two weeks and a right radical mastoidectomy was performed. The patient improved rapidly and the clinical picture of the patient resolved completely in 14 days. A control imaging for the resolution of thrombosis could not be performed because the patient was lost during follow-up.

## 3. Discussion

The causative agent of Lemierre's syndrome is commonly* Fusobacterium necrophorum*, which is an anaerobic bacteria present in the oral cavity, gastrointestinal system (GIS), and female genital system flora. In the last 20 years, an increase in its incidence has been reported owing to various reasons. The disease may be seen in any age group; however, more than 70% of cases are healthy adolescents and young adults. It has been reported more commonly in males than females [4, 5]. An association between Lemierre's syndrome and diabetes mellitus has been reported [6] in the literature as well.

Prior to the disease, in general as in the current case, an oropharyngeal disease history of about one week is present. At the beginning of the disease, the physical examination findings may be very slight [4, 7]. Typically, the clinical course shows a gradual, step-by-step pattern in LS. The preliminary period includes findings such as fever, pharyngitis, otitis media, mastoiditis, and parotitis. Later, local microbial invasion to the lateral pharyngeal area and extension to the IJV by the way of infected peritonsillary veins and lymphatics may be determined. Owing to the involvement of the posterior compartment, cranial nerve X and XII palsies and Horner syndrome may also develop. Lastly, bacteremia and septic emboli may be seen in the other organs like lungs, bones, brain, and liver [4, 7, 8]. In the present case, transition to the last step was not yet present, but OM, mastoiditis, and thrombophlebitis of the IJV and sigmoid-transverse sinus were present.

Central nervous system involvement is extremely rare in LS, but purulent meningitis, cerebral abscess, and extension of IJV thrombophlebitis to the sigmoid and cavernous sinuses in a retrograde manner have been reported [5, 9]. Though few in number, similar to the current case, IJV thrombophlebitis and sigmoid and transverse sinus thrombosis cases secondary to OM and mastoiditis have been reported in the literature [7].

In the diagnosis of LS, radiology is tremendously important. Doppler US considerably enables the demonstration of the thrombus in IJV without ionizing radiation. In regions to which US cannot extend, such as the skull base or below the clavicle, contrasted CT and MRI warrant complete IJV visualization and moreover enable the visualization of anterograde and retrograde extensions and accompanying complications, as in the current case [8]. Since we have thought of retropharyngeal abscess in our case, contrasted CT and MRI investigations were performed. The CT and MRI revealed right IJV thrombophlebitis findings, and filling defects in sigmoid-transvers sinus and air bubbles in IJV and transverse sinus were thought to be due to anaerobic infection. Although many LS cases were defined in the literature, air bubbles were not reported in the cranial sinuses or IJV in any of the cases. Interestingly, in the present case, there were air bubbles both in the transverse sinus and in the IJV. Radiology is also effective in determination of metastatic infections [8].

Satisfactory results are obtained in most cases with antimicrobial treatment and surgical drainage. The use of anticoagulants in LS is controversial due to a lack of controlled studies [10]. We did not use anticoagulants in the present study. We could not obtain control imaging and, therefore, cannot comment. However, with radical mastoidectomy and appropriate antibiotic treatment, the patient completely recovered.

Since the incidence of Lemierre's syndrome has increased over the last 20 years and the disease has high mortality and morbidity rates, prompt diagnosis of the disease is important. Using radiological instruments in the diagnosis, such as Doppler US, contrasted CT, and/or MRI should not be delayed in suspected cases.
